# Kynurenine Pathway Metabolites in Humans: Disease and Healthy States

**DOI:** 10.4137/ijtr.s2097

**Published:** 2009-01-08

**Authors:** Yiquan Chen, Gilles J. Guillemin

**Affiliations:** 1 School of Medical Sciences, University of New South Wales, Sydney 2052, Australia; 2 St. Vincent’s Centre for Applied Medical Research, Darlinghurst 2010, Australia

## Abstract

Tryptophan is an essential amino acid that can be metabolised through different pathways, a major route being the kynurenine pathway. The first enzyme of the pathway, indoleamine-2,3-dioxygenase, is strongly stimulated by inflammatory molecules, particularly interferon gamma. Thus, the kynurenine pathway is often systematically up-regulated when the immune response is activated. The biological significance is that 1) the depletion of tryptophan and generation of kynurenines play a key modulatory role in the immune response; and 2) some of the kynurenines, such as quinolinic acid, 3-hydroxykynurenine and kynurenic acid, are neuroactive. The kynurenine pathway has been demonstrated to be involved in many diseases and disorders, including Alzheimer’s disease, amyotrophic lateral sclerosis, Huntington’s disease, AIDS dementia complex, malaria, cancer, depression and schizophrenia, where imbalances in tryptophan and kynurenines have been found. This review compiles most of these studies and provides an overview of how the kynurenine pathway might be contributing to disease development, and the concentrations of tryptophan and kynurenines in the serum, cerebrospinal fluid and brain tissues in control and patient subjects.

## Introduction

Tryptophan is one of the 9 essential amino acids that the human body is incapable of synthesizing and thus, has to be obtained through external sources. Once absorbed by the body, tryptophan travels around the periphery circulation either bound to albumin or in free form, the two states existing in equilibrium, with the former accounting for up to 90%.^1^ However, tryptophan can only be transported across the blood brain barrier in its free form by the competitive and non-specific L-type amino acid transporter.^2^ Once in the central nervous system (CNS), tryptophan acts as a precursor to various metabolic pathways. This versatility results in different end-products, such as protein, serotonin and kynurenines.^3^ In both the peripheral and central systems, the kynurenine pathway represents a major route for the metabolism of tryptophan.

**Figure 1.**
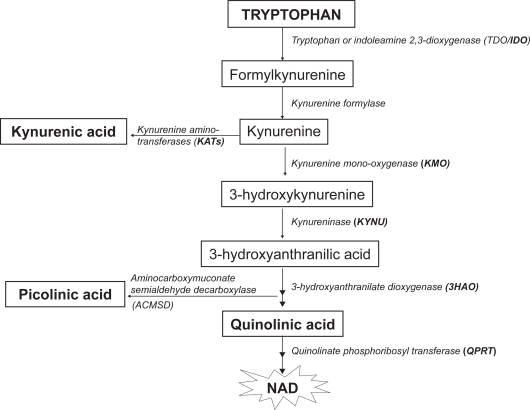
A schematic diagram of the kynurenine pathway.

Following the kynurenine pathway (Fig. 1), tryptophan is oxidized by cleavage of the indole-ring, initiated either by tryptophan 2,3-dioxygenase (TDO), indoleamine 2,3-dioxygenase 1 (IDO-1) or IDO-2, a newly discovered IDO related enzyme.^4^–^7^ TDO resides primarily in the liver and is induced by tryptophan or corticosteroids.^4^ IDO-1, on the other hand, is the predominant enzyme extra-hepatically and can be found in numerous cells, including macrophages, microglia, neurons and astrocytes.^8^–^11^ It is up-regulated by certain cytokines and inflammatory molecules, such as lipopolysaccharides, amyloid peptides and human immunodeficiency virus (HIV) proteins,^5^,^12^,^13^ but its most potent stimulant is interferon gamma (IFN-γ).^14^,^15^ IFN-γ is able to induce both the gene expression and enzymatic activity of IDO-1.^16^,^17^ Recently, an IDO related enzyme, IDO-2, was identified.^7^,^6^ The encoding genes for IDO-1 and IDO-2 are located next to each other and IDO-2 possesses similar structural and enzymatic activities as IDO-1. However, IDO-2 differs in its expression pattern and signalling pathway and is preferentially inhibited by D-1-methyl-tryptophan.^7^,^6^

As tryptophan proceeds along the kynurenine pathway to achieve the final product, nicotinamide adenosine dinucleotide (NAD), kynurenine is the first stable intermediate formed. Subsequently, several neuroactive intermediates are generated. These comprise the free-radical generator, 3-hydroxyanthranilic acid,^18^ the excitotoxin and N-methyl-D-aspartic acid (NMDA) receptor agonist, quinolinic acid,^19^ the NMDA antagonist, kynurenic acid,^20^ and the neuroprotectant, picolinic acid.^21^

During an immune response, the release of IFN-γ by activated T cells and leukocytes leads to an accelerated and sustained degradation of tryptophan. This significance was first speculated to be a defence mechanism that starved tumour cells, pathogens and parasites of tryptophan.^22^,^23^ However, with the discovery that IDO-1 activity was necessary for the preservation of allogenic fetuses in mice, further *in vitro* research found that tryptophan depletion had an anti-proliferative and apoptotic effect on T cells.^24^–^26^ In particular, the general control non-derepressible-2 (GCN2) kinase was identified as a key mediator in IDO-1 induced tryptophan depletion immunosuppression.^27^ The activation of GCN2 triggers a stress-response program that can result in cell-cycle arrest, differentiation, adaptation or apoptosis.^28^–^30^ Furthermore, some of the kynurenines, such as quinolinic acid and 3-hydroxyanthranilic acid, can also effectively suppress T cell proliferation.^31^ This inhibition appears to selectively target immune cells undergoing activation 32 and these kynurenines may act in concert to produce an additive effect.^33^ Lastly, the production of the excitotoxin quinolinic acid is often significantly increased following inflammation and resulting immune activation.^34^

To date, the kynurenine pathway has been implicated in a variety of diseases and disorders, including acquired immune deficiency syndrome (AIDS) dementia complex, Alzheimer’s disease (AD), schizophrenia, Huntington’s disease, amyotrophic lateral sclerosis (ALS) and neoplasia,^35^–^43^ and numerous studies have measured the levels of tryptophan and kynurenines under those conditions. Significant imbalances in tryptophan and its metabolites were frequently observed, which when brought back within normal ranges, often resulted in alleviation of symptoms. This review brings together most of these studies to provide a better idea of the expected differences in tryptophan and kynurenine levels in the serum, cerebrospinal fluid (CSF) and brain between disease and healthy states.

## The Kynurenines

### Kynurenic acid

Kynurenic acid is an endogenous neuroprotectant that is usually present in the brain at nanomolar concentrations.^44^ An antagonist to quinolinic acid, kynurenic acid acts on the glycine modulatory site of the NMDA receptor at low concentrations;^45^ and at higher concentrations, at the glutamate site of the NMDA receptors and also on the a-amino-3-hydroxy-5-methyl-4-isoxazolepropionate (AMPA) receptors.^46^ In addition, it also antagonizes the alpha 7 nicotinic acetylcholine receptors^47^ and selectively activates a G-protein coupled receptor, GPR35.^48^

Increases in brain kynurenic acid were first observed to have sedative and anticonvulsant effects.^49^ Later, it was found to be protective against brain ischemia.^50^ The elevation in CSF kynurenic acid in schizophrenic patients also provided a new insight into the possible effect of kynurenic acid on the glutamatergic and dopaminergic systems, and its potential role in the pathogenesis of schizophrenia.^51^,^52^ Although it is argued that the physiological levels of kynurenic acid may fall below that which is necessary for glutamate receptor antagonism, at specific sites within synapses, those levels may be sufficient.^53^ This hypothesis is supported by the significant reduction in glutamate release and extracellular levels of dopamine seen with kynurenic acid in rats *in vivo.*^54^,^55^ In addition, the use of kynurenine 3-hydroxylase inhibitor also led to a hyperactivity in dopamine neurons.^56^

In a septic shock mouse model, kynurenic acid was able to significantly decrease the release of tumour necrosis factor α (TNF-α), nitric oxide and high mobility group box 1 protein, a molecule likely to be involved in lipopolysaccharides mediated toxicity.^57^,^58^ Rather unexpectedly though, kynurenic acid inhibited the release of fibroblastic growth factor 1, a compound that supports growth and recovery of injured cells and enhances proliferation of glia cells.^59^ However, this does not necessarily challenge the concept of kynurenic acid being neuroprotective but definitely warrants more investigation.

### 3-hydroxyanthranilic acid

3-hydroxyanthranilic acid can be derived either from the hydrolysis of 3-hydroxykyurenine or the oxidation of anthranilic acid (Fig. 1). Besides playing a role in immunoregulation,^60^–^62^ 3-hydroxyanthranilic acid is also a neurotoxin. Intracerebral injection of 3-hydroxyanthranilic acid leads to a decrease in choline acetyltransferase activity similar to those seen with quinolinic acid, but to a lesser extent.^21^ In addition, it is a free radical (superoxide and hydrogen peroxide) generator in the presence of copper.^18^ However, 3-hydroxyanthranilic acid can also act as an antioxidant, scavenging peroxyl radicals more effectively than equimolar concentrations of either ascorbic acid or Trolox (a water soluble analogue of vitamin E).^63^

In murine macrophages, 3-hydroxyanthranilic acid at sub-millimolar concentrations can inhibit the activation of nuclear factor κB and likewise, the expression and activity of inducible nitric oxide synthase (iNOS).^64^ iNOS catalyses the formation of NO, which is strongly correlated with antimicrobial and antitumoral activities in mouse macrophages.^65^ Following along the lines of tumoregenesis, non-toxic concentrations of 3-hydroxyanthranilic acid has no effect on T cell receptor triggered CD8^+^ T lymphocyte proliferation, but greatly inhibits that induced by antigen-independent cytokine (particularly interleukin (IL)-2, IL-7 and IL-15) stimulation.^66^ Thus, in the context of cancer, tumour cells could severely arrest CD8^+^ T cell proliferation by driving cytokine production without effectively triggering T cell receptor response.^66^

Furthermore, 3-hydroxyanthranilic acid exerts a selective apoptotic effect on murine thymocytes and T helper 1 (Thl) cells via the activation of caspase-8 and release of cytochrome *c* from mitochondria, but independent of the Fas pathway.^61^ This action occurs at concentrations well below those resulting in neurotoxicity or apoptosis of macrophages and could represent an important role in peripheral immunoregulation.^61^ Adding to this, following antigen stimulation of myelin basic protein Acl-11 T cell receptor transgenic CD4^+^ T cells, 3-hydroxyanthranilic acid was associated with a Gi/S phase arrest in CD4^+^ T cells and a cytokine profile shift in favour of Th2 cells.^67^ This finding has important implications in the treatment of multiple sclerosis (MS).^67^

### Picolinic acid

Picolinic acid, a monocarboxylic acid, is an endogenous neuroprotectant and a natural iron and zinc chelator.^21^ It controls cellular growth and has anti-tumoral, antifungal and antiviral activities. *In vitro*, picolinic acid arrests normal cells in G_1_ phase, possibly through the interactions with NAD^+^ as the inhibition can be overcome by nicotinamide.^68^ Recently, the characterization of the kynurenine pathway in human primary adult neurons and SK-N-SH neuroblastoma cell line found the former capable of synthesizing picolinic acid but not the latter.^69^ This variation in kynurenine pathway activation in neuroblastoma cells may provide a key to understanding tumour persistence and associated neurotoxicity.

*In vivo*, the antitumoral effect of picolinic acid was observed when treatment in mice inoculated with MBL-2 lymphoma cells altered their ribosomal ribonucleic acid (RNA) metabolism, augmenting the cytotoxic and tumoricidal activities of macrophages, resulting in increased survival rate.^70^,^71^ As an antifungal, picolinic acid acts synergistically with IFN-γ to amplify the inhibitory effect of neutrophils, inhibiting *Candida albicans* growth *in vitro* and *in vivo*.^72^,^73^ Although the mechanism of this co-stimulatory effect is unclear, it is known to be vulnerable to IL-4 suppression.^74^ In mouse, the synergy with IFN-γ is further extended to include NOS and TNF-α gene expression.^75^,^76^

At relatively high concentrations (1.5–3 mM), picolinic acid exerts antiviral, cytotoxic and apoptotic effects on HIV-1 and human herpes simplex virus-2,^77^ which is likely to be associated with an up-regulation in macrophage inflammatory protein (MIP)-1α and MIP-1β messenger RNA (mRNA) expression, as both compounds inhibit HIV-1 infection.^78^–^80^ Interestingly, this stimulatory effect on MlP-lα and β is antagonized by IFN-γ.^81^ The complex interplay between picolinic acid and IFN-γ highlights the importance of these molecules on the regulation of macrophage activities and perhaps, the inflammatory response.^81^

Like kynurenic acid, picolinic acid blocks quinolinic acid induced neurotoxicity, but not the neuroexcitatory component.^21^,^82^ Compared to kynurenic acid though, picolinic acid is less potent and appears to act via a different mechanism, attenuating calcium dependent glutamate release and/or chelating endogenous zinc.^83^,^84^,^85^ This lower potency of picolinic acid may also be partly explained by the weak stimulatory action it has on glutamate release from the striatum.^84^

### Quinolinic acid

Quinolinic acid is a heterocyclic amino acid that selectively activates the neuronal NMDA subtype of glutamate receptors.^19^ Within the brain, quinolinic acid concentrations are normally lower compared to blood and systemic tissues as tryptophan is metabolized to 5-hydroxytryptamine rather than to formylkynurenine.^86^ However, during an immune response, either systemic or central, IDO-1 activity and levels of quinolinic acid rise dramatically, the significance of which is still obscure.^87^–^89^

Under inflammatory conditions in the brain, infiltrating macrophages, microglia and dendritic cells are major sources of quinolinic acid production.^90^,^91^,^92^ Astrocytes, in contrast, are incapable of synthesizing quinolinic acid due to the absence of the enzyme, kynurenine hydroxylase.^93^ Rather, both astrocytes and neurons,^9^ being neuroprotective, uptake quinolinic acid and catabolize it to NAD. However, this catabolic system is easily saturated in the presence of high amounts of quinolinic acid, produced under pathological conditions, resulting in the toxic accumulation of quinolinic acid within the cells.^94^

As an endogenous molecule of the mammalian CNS, the immune and neurotoxic properties of quinolinic acid are of special interest.^95^ *In vitro*, the synthesis of quinolinic acid by CD8^−^” dendritic cells induced apoptosis in Thl target cells;^96^ and quinolinic acid can also selectively inhibit the proliferation of CD4^+^ and CD8^+^ T lymphocytes and natural killer cells undergoing activation, the effect of which is amplified in the absence of tryptophan.^32^

In direct intracerebral administration and neuronal cell cultures, quinolinic acid led to neuronal death.^97^,^98^ Similarly, the chronic exposure to sub-micromolar concentrations of quinolinic acid on neurons produced an adverse effect and the converse was true too.^99^,^98^ *In vivo*, injection of quinolinic acid into discrete regions of the rat brain caused axon-sparing lesions similar to those produced by kainic and ibotenic acid.^97^ Several studies have already provided strong evidence suggesting that quinolinic acid plays a significant pathological role in the development of neurodegenerative disorders, such as Huntington’s disease (HD),^99^ AD^100^,^101^,^102^ and AIDS dementia complex.^103^,^104^,^105^

## The Kynurenine Pathway in Disease States

Under various pathological conditions, an accelerated degradation of tryptophan with an accompanying increase in kynurenines is often observed in the serum, CSF and/or brain tissue (Tables 1, 2 and 3). Moreover, the breakdown of tryptophan via the kynurenine pathway is often routed preferentially towards the production of quinolinic acid. The pathologies associated with the up-regulation of the kynurenine pathway include infectious diseases (e.g. HIV), neurological disorders (e.g. AD, HD and ALS), affective disorders (e.g. schizophrenia, depression and anxiety), autoimmune diseases (e.g. MS and rheumatoid arthritis), peripheral conditions (e.g. cardiovascular disease) and malignancy (e.g. haematological neoplasia and colorectal cancer). However, significant elevations in tryptophan levels in lung and breast cancer have also been reported.^106^

We also observed an increase in tryptophan levels ALS patients’ samples (unpublished). At this stage, we speculate that this phenomenon might be associated with either a disturbance in albumin binding of tryptophan, an over-compensatory response to decreased tryptophan concentrations in the brain and/or a malfunctioning in the L-type amino acid transporter at the blood brain barrier in ALS. The elevation in tryptophan notwithstanding, ALS patients still exhibited a larger kynurenine/tryptophan (K/T) ratio, an index for IDO activity, than control subjects due to a significant concomitant rise in kynurenine.

The enhanced degradation of tryptophan and higher K/T ratio are also often associated with advanced stages of disease, more severe symptoms or a fatal outcome.^107^ ^108^,^109^ However, it is important to note that a progressive increased in tryptophan catabolism is part of the “normal” ageing process.^110^ Nonetheless, the degree of tryptophan depletion is still far more substantial in neurodegenerative disorders compared to normal ageing and most of the studies on pathological conditions were performed using age matched control subjects.^111^,^100^

In some studies, neopterin concentrations were also measured. Neopterin is a marker for immune activation and show a correlation with the K/T ratio and kynurenine, and inversely with tryptophan.^112^,^113^,^87^ This suggests an increase in endogenous IFN-γ production and an up-regulation in the kynurenine pathway. Indeed, HIV patients exhibit a 10-fold increase in IFN-γ through direct measurements.^114^

When HIV patients are treated with highly active antiretroviral therapy (HAART) or antiretroviral treatment (ART), which significantly decreases immune activation through reduction in viral load, a repletion in tryptophan and reduction in kynurenine and quinolinic acid often follows.^115^,^116^ It is interesting to note that the alteration in tryptophan levels occurred in the absence of any dietary modification and that changes in K/T ratio correlated strongly with HIV mRNA and CD4^+^ T cell count.^116^

The most important consequences of dramatic decline in tryptophan, thus, are likely to be immunosuppression and immunodeficiency, particularly evident in HIV infection, but also in autoimmune diseases and cancer. Other effects include weight loss, mood disturbances and cognitive impairment.^117^,^118^

In *anorexia nervosa*, underweight anorexic patients had lower tryptophan levels which rose with weight normalization.^117^ The association of tryptophan levels and the development of cachexia and weight loss are also evident in neoplasia.^119^,^120^ This could be associated with the release of pro-inflammatory cytokines. TNF-α, for instance, is a known cachexia, featuring prominently in muscle pathophysiology.^121^ The heightened catabolism of tryptophan via the kynurenine pathway may also divert this essential amino acid away from protein synthesis, thus, contributing to weight loss and muscle wasting.^119^

Tryptophan also acts as a precursor for the synthesis of serotonin, which has a broad spectrum of action, two of which are in mood and cognitive functioning.^118^,^122^ Imbalances in kynurenines and significant decline in 5-hydroxyindoleacetic acid (5-HIAA), a serotonin metabolite, have been reported in major depression, MS and cardiovascular disease, among others.^123^,^124^,^125^ However, the activation of the immune response is also postulated as a cause of depression^126^,^127^ and a strong association exists between inflammatory diseases and depression.^128^,^129^

In normal subjects, the deliberate depletion of tryptophan selectively impaired long-term memory consolidation,^130^ opposed to the results observed with the administration of selective serotonin reuptake inhibitors.^131^ In AD and HD patients, the K/T ratio was also inversely correlated with cognitive performance;^132^,^133^ and in HIV-1 patients, treatment with HAART, which elevates tryptophan levels, markedly improved cognitive function.^134^,^135^

## Potential Treatments Involving the Kynurenine Pathway

**Figure 2.**
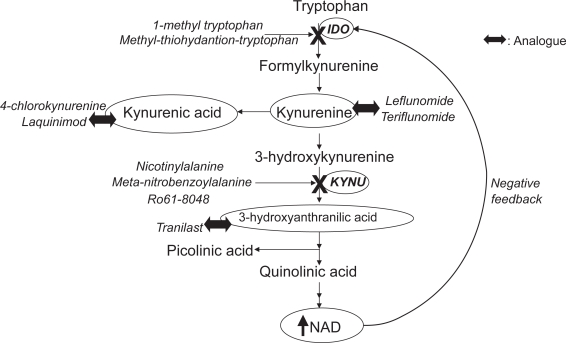
Drugs targeting the kynurenine pathway—inhibitors and analogues.

The involvement of the kynurenine pathway in a wide range of diseases suggests that research on treatment strategies targeting the kynurenine pathway (Fig. 2) may provide an alternative means of treatment or as a complement to what is already available.

### Niacin supplementation

One of the consequences of accelerated degradation and depletion of tryptophan in the body is the suppression of T cell proliferation,^136^ which compromises the body’s immunity. Repletion of tryptophan could lead to improve immune response but may also inadvertently cause an increase in neurotoxins. Niacin supplementation, however, provides an indirect way to increase tryptophan and act as a feedback mechanism to suppress IDO-1 activity.^137^ In clinical studies, dietary supplementation of niacin to HIV-1 patients was associated with higher CD4 counts and improved survival rates.^138^,^139^

### IDO inhibitors

The suppression of IDO-1 activity has been targeted directly in cancer research. Using transgenic mouse model of breast cancer, IDO-1 inhibitors, 1-methyl-DL-tryptophan and methyl-thiohydantoin-tryptophan, were able to potentiate the efficacy of chemotherapy drugs, promoting tumour regression without increasing the side effects.^140^ The discovery of the preferential inhibition by D-l-methyl-tryptophan on IDO-2 could also provide the key to understanding the mechanism behind the antitumoral action of 1-methyl-tryptophan and in designing future IDO inhibitors.^7^

### Kynurenine analogues

Another approach to modifying the kynurenine pathway is to skew the balance of kynurenines towards neuroprotection and away from neurotoxicity. Currently, there are several therapeutic agents, either already on the market or undergoing clinical trials, which are either analogues of neuroprotective kynurenines or act to inhibit the production of quinolinic acid. They include 4-chlorokynurenine, laquinimod, leflunomide, tranilast, nicotinylalanine, meta-nitrobenzoylalanine and Ro61-8048.

7-chlorokynurenate, a synthetic derivative of kynurenic acid, attenuates the neurotoxic effect of quinolinic acid through blockade of the glycine modulatory site of the NMDA receptor.^141^,^142^ However, 7-chlorokynurenate crosses the blood brain barrier with great difficulty.^143^ 4-chlorokynurenine, a precursor of 7-chlorokynurenate, on the other hand, is able to overcome this obstacle.^144^ When administered together with quinolinic acid *in vivo*, 4-chlorokynurenine was converted into the active 7-chlorokynurenate successfully, providing neuroprotection.^145^,^146^

Laquinimod (ABR-215062), a novel synthetic quinoline, has demonstrated immunomodulatory properties without immunosuppression in preclinical trials.^147^–^149^ In MS animal model, experimental autoimmune encephalomyelitis (EAE), laquinimod delayed disease progression, inhibited infiltration of CD4^+^ T cells and macrophages into the CNS and modulated the immune response in favour of Th2/Th3 cytokines IL-4, IL-10 and transforming growth factor (TGF-P).^150^ Furthermore, in patients with relapsing MS, treatment with laquinimod successfully reduced the development of active lesions.^151^

Leflunomide (Avara^®^), an immunosuppressive and anti-inflammatory prodrug is converted into terflunomide *in vivo* (A771126). Terflunomide is an inhibitor of mitochondrial dihydroorotate dehydrogenase, an essential enzyme for *de novo* pyrimidine synthesis.^152^ In 1998, the Food and Drug Administration (FDA, U.S.A.) approved leflunomide for the treatment of rheumatoid arthritis. Furthermore, in a recent phase II trial with MS patients, terflunomide proved well tolerated and effective in reducing active lesions.^153^

Tranilast (Rizaben^®^), a synthetic anthranilic acid derivative drug, has the ability to inhibit the release of chemical mediators, TGF-É¿ and suppress angiogenesis.^154^,^155^ Tranilast has been effective against many diseases, such as allergic rhinitis, atopic dermatitis and bronchial asthma. Recently, when tested against EAE, tranilast inhibited the actions of Thl cells while enhancing those of Th2 cells, an action similar to that of natural tryptophan catabolites, 3-hydroxyanthranilic acid and 3-hydroxykynurenic acid.^67^

Finally, kynurenine hydroxylase inhibitors are also effective in diverting the kynurenine pathway away from the synthesis of quinolinic acid towards that of kynurenic acid. These compounds include nicotinylalanine, meta-nitrobenzoylalanine and Ro61-8048.^156^ Nicotinylalanine, an analogue of kynurenine, protects the brain from induced seizures^157^,^158^ and quinolinic acid induced striatal damage in the rat.^159^ With meta-nitrobenzoylalanine, sedation and anticonvulsant effects were achieved in rats,^160^ while reduced neuronal loss from brain ischemia were seen in gerbils.^50^ In immune activated mice, meta-nitronemzoylalanine also significantly decreased quinolinic acid production in the blood and brain.^161^ With Ro61-8048, there is an additional benefit of reducing glutamate levels in the extracellular spaces of the basal ganglia in rats, while maintaining the learning and memory process.^162^ In EAE rats, administration of Ro61-8048 significantly reduced the neurotoxic levels of 3-hydroxykynurenine and quinolinic acid in the CNS;^163^ and in a cerebral malaria mouse model, it significantly increased the neuroprotective levels of picolinic acid, prevented the development of neurological symptoms and prolonged survival by threefold.^164^ Like meta-nitrobenzoylalanine, Ro61-8048 too decreased neuronal loss due to brain ischemia.^50^

## Conclusion

The kynurenine pathway is an effective mechanism in modulating the immune response and in inducing immune tolerance. This is achieved by accelerating the degradation of tryptophan and the generation of kynurenines. The metabolites of the pathway, with their different inherent properties, can also synergize or antagonize the effects of one another. By measuring the levels of tryptophan, kynurenines and the K/T ratio under various pathological conditions, the degree of immune activation and the relationship between the kynurenine pathway and disease states may be gleaned. However, much research is still needed to fully understand the complex interaction between tryptophan, IDO and kynurenines among themselves and within the CNS and in the periphery. With the seemingly prevalent involvement of the kynurenine pathway in a wide range of different diseases and disorders, the knowledge gained from research focusing on the kynurenine pathway may be translated into designing novel and more effective treatment strategies.

## Figures and Tables

**Table 1.** Studies investigating kynurenine metabolites in plasma/serum.

**References**	**Pathology**	**Compound**	**Patients**	**Controls**	**Comments**
Werner et al. 1988^165^	HIV	TRP (μM)	44.8 ± 8.4^++^	91.0 ± 22.0	Neopterin levels were significantly increased in patients (39.1 ± 17.0 nM *vs.* 4.5 ± 1.5 nM).
		KYN (μM)	3.53 ± 0.89***	2.31 ± 0.77	
		T/K ratio	13.4 ± 3.7^++^	42.5 ± 13.7	
Larsson et al. 1989^166^	HIV	TRP (μM)	28.4	39.7	Platelets bound serotonin (5-HT) (ng/10^9^) significantly reduced in patients compared to controls (430 *vs.* 676).
Cascino et al. 1991^106^	Cancer	TRP (μM)	10.9 ± 5.2* (L-pre)	4.7 ± 0.7	L-pre: Lung cancer, pre-operation; B: Breast cancer; pt: post-operation; ^A^:P < 0.05 from pre-op. TRP data here is that of free tryptophan. Total plasma TRP was similar between patients and controls, pre-operation and post-operation.
			6.6 ± 3.2* (B-pre)		
			7.1 ± 2.6*^A^ (L-pt)	5.4 ± 0.9	
			4.6 ± l.l^A^ (B-pt)		
Fuchs et al. 1991^114^	HIV	TRP (μM)	57.0 ± 2.8** (+)	91.0 ± 6.63	IFN-γ (U/I): 259 ± 70** in seropositive patients compared to 23.5 ± 1.7 in seronegative patients.
		KYN (μM)	3.45 ± 0.14** (+)	2.31 ± 0.23	
Denz et al. 1993^120^	Hematological neoplasias	TRP (μM)	56.4 ± 13.1 (HD)	≤65	HD: Hodgkin’s disease; NHL: non-Hodgkin’s lymphoma; M/M: multiple myeloma/monoclonal gammopathy of unknown significance. An inverse correlation was found between TRP and weight loss in patients.
			50.5 ± 16.9^+^ (NHL)		
			44.9 ± 12.9^+^ (MM)		
		KYN (μM)	2.3 ± 1.1 (HD)	≤3.5	
			2.8 ± 1.4 (NHL)		
			2.5 ± 1.0 (M/M)		
Heyes et al. 1994^167^	Epilepsy (intractable complex partial seizure)	TRP (μM)	85.2 ± 3.7* (I.I.)	76.7 ± 4.7	I.I.: inter-ictal; P.I.: post-ictal Data are shown only when differences were significant. Patients’ data are approximates as results were presented only with a bar graph.
		KYN (μM)	68.5 ± 3.7^+^ (I.I.)	3.27 ± 0.3	
			70.4 ± 3.7^+^ (P.I.)		
		KYNA (nM)	55.6 ± 5.56^+^ (I.I.)	32.1 ± 3.6	
			60.2 ± 7.4l** (P.I.)		
		3-HK (nM)	No difference	383 ± 24	
		QUIN (nM)	73.1 ± 3.7*** (I.I.)	432 ± 60	
			70.4 ± 3.7*** (P.I.)		
Orlikov et al. 1994^168^	Anxiety (A) and Depression (D)	KYN (μM)	9.32 ± 0.2*** (A)	4.32 ± 0.3	After treatment, the KYN concentrations returned back to normal. A significant correlation exists between KYN concentrations and anxiety severity.
			2.98 ± 0.01* (D)		
Fujigaki et al. 1998^169^	None	KYN (μM)		1.6 ± 0.1	Species (human, macaques, rabbit, guinea pig, rat, gerbil and mouse) differences present in KYN and AA.
		AA (nM)		16.5 ± 0.7	
Heyes et al. 1998^170^	HIV	QUIN (nM)	16847 ± 3358**	451 ± 78	
Huengsberg et al. 1998^171^	HIV	TRP (μM)	33.2	56.3	Asym: asymptomatic patients. KT ratio (×1000): 119.9 in patients; 50.5 in asymptomatic AIDS subjects; 34.9 in controls. K/T ratio had a reciprocal relationship with CD4^+^ count.
			50.1 (asym)		
		KYN (μM)	3.98	1.98	
			2.55 (asym)		
Look et al. 2000^115^	HIV	TRP (μM)	44.6 (pre)	52.6	pre: pre-treatment with HAART. Post-treatment saw a significant increase in TRP and a decrease in QUIN.
		KYN (μM)	4.1*** (pre)	2.7	
		KYNA (μM)	27 (pre)	30.1	
		QUIN (nM)	848^+^ (pre)	303.3	
		K/T (×10^3^)	108.2*** (pre)	51.4	
Murr et al. 2001^112^	*Streptococcus pyogenes*	TRP (μM)	25.3** (STSS)		STSS: streptococcal toxic shock syndrome; data are median values. Neopterin levels: STSS (152 nM) *vs.* tonsillitis (12 nM). Neopterin levels correlated with kynurenine, K/T and inversely with tryptophan significantly.
			80.9 (tonsillitis)		
		KYN (μM)	12.8** (STSS)		
			2.7 (tonsillitis)		
		K/T (×10^3^)	560** (STSS)		
			40 (tonsillitis)		
Murray et al. 2001^137^	HIV	TRP (μM)	49.4 ± 6.5 (pre)		pre: pre-treatment; post: post-treatment. Treatment with 3 g of nicotinamide daily for 2 mths.
			69.2 ± 6.3 (post)		
(Zangerle et al. 2002)^116^	HIV	TRP (μM)	44.1 ± 13.3 (pre)	65.8 ± 12.8	pre: pre-treatment with ART. 6 mths after ART, median increase in TRP was 20.2%, median decrease in KYN was 19.3% and median decrease in KT ratio was 28.1%. During ART, change in KT ratio significantly correlated with change in HIV RNA, CD4^+^ T cells and neopterin.
		KYN (μM)	3.01 ± 0.91 (pre)	2.02 ± 0.66	
		K/T (×10^3^)	79.2 ± 60.3 (pre)	30.7 ± 8.7	
(Huang et al. 2002)^172^	Colorectal cancer	TRP (μM)	53.5* (median)	63.7	
		KYN (μM)	2.1 (median)	2.0	
		K/T (×10^3^)	42.9*	31.8	
(Ilzecka et al. 2003)^173^	ALS	KYNA (nM)	57.8 ± 35.0	59.6 ± 20.5	m/s.c.s.: mild/severe clinical status ^a^: significantly lower KYNA in s.c.s. compared to m.c.s. There was no difference in serum KYNA and type of ALS onset.
			81.6 ± 41.2^a^ (m.c.s.)		
			39.9 ± 14.7* (s.c.s.)		
Schrocksnadel et al. 2003^174^	Rheumatoid arthritis	TRP (μM)	44.95** (median)	62.62	
		KYN (μM)	1.86 (median)	2.06	
		K/T (×10^3^)	42.39**	31.72	
Wirleitner et al. 2003^175^	Coronary heart disease	TRP (μM)	53.5 ± 9.26**	65.9 ± 12.7	Subdividing patients into 3 groups: 1, 2/3-artery disease and those with restenosis showed no significant difference in TRP or KYN between groups.
		KYN (μM)	l.88 ± 0.53	l.85 ± 0.51	
		K/T (×10^3^)	36.3 ± 13.0**	28.l ± 5.15	
Schrocksnadel et al. 2005^176^	Gynaecological cancer	TRP (μM)	43.5* (median)	53.5	Subdivision of patients found only those with ovarian cancer had significantly lower TRP than control. TRP, KYN or K/T did not correlate with disease stage.
		KYN (μM)	1.91 (median)	1.73	
Stoy et al. 2005^42^	HD		Data in graphs:		The comparisons here are for baseline values only. The paper also looked at values after TRP depletion and loading. Big variations in QUIN values were observed but overall, the concentrations were similar between patients and controls. Neopterin levels were significantly increased in patients (18.6 ± 1.7 nM *vs.* 12.7 ± 0.8 nM).
		TRP (μM)	No difference		
		KYN (μM)	Higher**		
		KYNA (μM)	No difference		
		3-HK (μM)	Lower*		
		3-HAA (μM)	Lower*		
		QUIN (μM)	No difference		
		K/T (×10^3^)	Higher**		
Forrest et al. 2006^177^	Osteoporosis	TRP (μM)	36.69 ± 1.8 (pre)	42.08 ± 2.28	Patients were treated for 2 yrs with either raloxifene or disodium etidronate with calcium.
			42.42 ± 1.65 (post)		
		KYN (μM)	1.87 ± 0.12 (pre)	1.96 ± 0.11	
			2.01 ± 0.14 (post)		
		KYNA (nM)	32.68 ± 2.98 (pre)	24.76 ± 2.46	
			34.09 ± 3.75 (post)		
		3-HAA (nM)	1.04 ± 0.13* (pre)	7.89 ± 1.15	
		AA (nM)	139 ± 14.7* (pre)	21.56 ± 2.25	
Mackay et al. 2006^178^	Chronic brain injury		Data in graphs:		The comparisons here are for baseline values only. The paper also looked at values after TRP depletion and loading. Big variations in QUIN values were observed but overall, the concentrations were similar between patients and controls. Neopterin levels were significantly increased in patients (18.8 ± 2.4 nM *vs.* 12.7 ± 0.8 nM).
		TRP (μM)	No difference		
		KYN (μM)	Higher*		
		KYNA (μM)	Lower**		
		3-HK (uM)	Lower**		
		3-HAA (μM)	Lower*		
		QUIN (μM)	No difference		
		K/T (×10^3^)	Higher**		
Darlington et al. 2007^179^	Stroke		Data in graphs:		The comparisons were made at different time points after stroke and the values here are only baseline values. Various correlations between kynurenines, neopterin, peroxidation products and volume of brain damage were analysed and TRP metabolism may contribute to brain damage following stroke.
		TRP (μM)	Lower^+^		
		KYN (μM)	Higher*		
		3-HAA (nM)	Lower^+^		
		AA (nM)	Higher**		
		K/T (×10^3^)	Higher^+^		
Hartai et al. 2007^180^	AD	KYN (μM)	2.5 ± 0.1	2.01 ± 0.2	In red blood cells, comparing patients to controls, KYNA (nM): 43.9 ± 5.9*vs. 67.4 ± 8.6; KYN (mM): 8.1 ± 0.5 *vs.* 9.3 ± 0.6. Activities of KAT I and II were similar in both instances in patients and controls.
		KYNA (nM)	15.82.31 ± 1.1*	23.13 ± 2.2	
Myint et al. 2007^123^	Major depressioin	TRP (μM)	65.8 ± 15.57	69.71 ± 13.65	
		KYN (\M)	1.81 ± 0.56	1.87 ± 0.43	
		KYNA (nM)	24.29 ± 8.09**	35.95 ± 13.4	
		3-HAA (nM)	24.53 ± 11.91	24.12 ± 7.3	
		K/T (×10^3^)	25 ± 12*	17 ± 14	
Schrocksnadel et al. 2006^181^	Rheumatoid arthritis	TRP (μM)	58.0 ± 19.3*		There was an inverse relation between TRP and the disease stage (*P* < 0.01)
		KYN (nM)	2.20 ± 0.82*		
Chen et al. unpublished^182^	ALS	TRP (μM)	143.28 ± 5.64^++^	75.0 ± 10.5	
		KYN (μM)	4.02 ± 0–2^++^	2.52 ± 0.19	
		QUIN (MM)	0.37 ± 0.018*	0.30 ± 0.026	
		PIC (MM)	1.42 ± 0.087*	2.38 ± 0.37	
		K/T (×10^3^)	37 ± 2.5	39 ± 4	

* *P* < 0.05;

** *P* < 0.01;

*** *P* < 0.005;

+ *P* < 0.001;

++ *P* < 0.0001.

**Table 2.** Studies investigating kynurenine metabolites in CSF.

**Ref.**	**Pathology**	**Compound**	**Patients**	**Controls**	**Comments**
Young et al. 1983^183^	Epilepsy	TRP (μM)	1.58 ± 0.61	1.66 ± 0.64	CSF data shown here were from the lumbar region. Cisternal CSF showed no differences between patients and controls and there were no CSF gradient differences either.
		KYN (nM)	28.4 ± 15.3*	43.9 ± 24.5	
		5-HIAA (nM)	96.7 ± 37.7	117.2 ± 62.7	
Larsson et al. 1989^166^	HIV	TRP (nM)	1518	2179	No significant change in 5-HIAA.
Baig et al. 1991^125^	MS and Cerebro-vascular disease (CVD)	TRP (nM)	1.25 ± 0.14^+^ (MS)	2.02 ± 0.34	Metabolites of the noradrenergic and dopaminergic systems [3-methoxy-4-hydroxyphenylglyco (MHPG), 3,4-dihydroxyphenylacetic acid (DOPAC) and homovanillic acid (HVA)] were also found to be significantly different in MS and CVD patients compared to controls.
			3.34 ± 0.54^+^ (CVD)		
		5-HT (pM)	5 ± 1* (MS)	7 ± 2	
			7 ± 2 (CVD)		
		5-HIAA (pM)	116 ± 15** (MS)	173 ± 20	
			299 ± 50** (CVD)		
Gisslen et al. 1994^184^	HIV	TRP (nM)	1097 (pre)		3–14 months treatment with zidovudin. Decrease in neopterin correlated with increase in TRP. 5-hydroxyindoleacetic acid (5-HIAA).
			1535.8 (post)		
Heyes et al. 1994^167^	Epilepsy (intractable complex partial)	TRP (μM)	No difference	1.32 ± 0.13	I.I.: inter-ictal; P.I.: post-ictal Data are shown only when differences were significant. Patients’ data are approximates as results were presented only with a bar graph. QUIN:KYNA in patients *vs.* controls: 61.1 ± 11.1** (I.I.), 58.3 ± 5.55***(P.I.) *vs.* 86.1 ± 19.4
		KYN (nM)	68.1 ± 2.78^+^ (I.I.)	42.2 ± 3.8	
			65.3 ± 2.78^+^ (P.I.)		
		KYNA (nM)	No difference	2.32 ± 0.35	
		QUIN (nM)	72.2.1 ± 2.78^+^ (I.I.)	21.9 ± 2.8	
			68.1 ± 2.78^+^ (P.I.)		
Demitrack et al. 1995^117^	Eating disorders (anorexia nervosa)	TRP (nM)	1.9 ± 0.5	2.1 ± 0.3	In anorectics, weight normalized restored all compounds tested to within the control range. The relative amount of QUIN (QUIN: KYNA) was significantly higher during the underweight phase for anorectics. Kynurenines were within control range for normal weight bulimics.
		KYN (nM)	25.6 ± 9.9	34.4 ± 12.3	
		KYNA (nM)	1.5 ± 0.5*	2.8 ± 1.2	
		QUIN (nM)	13.4 ± 5.4	13.8 ± 4.3	
		5-HIAA (nM)	107.2 ± 31.4*	146.3 ± 30.2	
Heyes et al. 1995^185^	CNS pathology	QUIN (nM)	31 ± 5 (Hy)	20 ± 2	Hy: hydrocephalus; H: haemorrhage; T: tumour; C: CSF infection. Subjects were all children. Both TNF-a and IL-6 were increased, with a significant correlation between IL-6 and QUIN.
			200 ± 113** (H)		
			282 ± 82** (T)		
			1084 ± 549** (C)		
		KYN (nM)	185 ± 40 (Hy)	54 ± 7	
			254 ± 128** (H)		
			1698 ± 589** (T)		
			2610 ± l 067** (C)		
Fujigaki et al. 1998^169^	None	KYN (nM)		29.1 ± 3.2	Species (human, macaques, rabbit, guinea pig, rat, gerbil and mouse) differences detected in levels of KYN and AA.
		AA (nM)		16.3 ± 4.2	
Heyes et al. 1998^170^	HIV	QUIN (nM)	3789 ± 888**	22.1 ± 2.1	
Erhardt et al. 2001a^51^	Schizophrenia	KYN (nM)	1.67 ± 0.027*	0.97 ± 0.07	A correlation between age and KYN was found in schizophrenics.
Medana et al. 2002^186^	Malaria (severe)	KYNA (μM)	0.06	0.07	None of the kynurenines were associated with convulsions or coma.
		QUIN (μM)	0.80^++^	0.07	
		PIC (μM)	0.19^+^	0.08	
Rejdak et al. 2002^187^	MS	KYNA (nM)	0.41** (MS)		MS: were patients with relapsing MS during remission or not progressing for at least 2 months; ID: infectious inflammatory disease; OND: non-inflammatory neurological disorders. MS had significantly lower KYNA than either ID or OND.
			0.67 (OND)		
			1.7 (ID)		
Ilzecka et al. 2003^173^	ALS	KYNA (nM)	2.41 ± 1.7 (grp)	1.59 ± 0.9	Bul: bulbar onset; s.c.s: severe clinical status No significant difference between KYNA levels and gender and no correlation between KYNA and age.
			3.61 ± 2.0** (bul)		
			3.26 ± 2.1*(s.c.s.)		
Medana et al. 2003^109^	Cerebral Malaria (Malawian children)	QUIN (μM)	0.09		For QUIN, KYNA and PIC, 72% (2%), 77% (43%) and 74% (38%) of Malawian children had higher levels than median (reference range) UK control levels respectively. Elevated levels of QUIN and PIC were associated with a fatal outcome. Other diseases tested include convulsions, sepsis and acute hepatitis.
		KYNA (μM)	0.21		
		PIC (μM)	0.18		
Nilsson et al. 2005^188^	Schizophrenia	KYNA (nM)	1.45 ± 0.10* (grp)	1.06 ± 0.06	Grp: All patients; 1st: Drug naive, first episode patients; T: patients undergoing treatment with anti-psychotic drugs; no D: patients who had been treated but are now drug free. In patients, a positive correlation was found between KYNA levels and age.
			1.53 ± 0.19* (1st)		
			1.53 ± 0.17*(T)		
			1.16 ± 0.06 (noD)		
Atlas et al. 2007^189^	HIV	KYNA (nM) median levels	4.54 (psy.) 3.02 (no psy.)	1.23	psy: psychotic symptoms In controls, KYNA levels were significantly higher in females (2.29 nM) than males (1.10 nM) *(P* < 0.05). However, this gender difference was absent in the patient population.
Chen et al. unpublished^182^	ALS	TRP (μM)	5.02 ± 0.19	2.58 ± 0.16	
		KYN (μM)	0.23 ± 0.0016	0.027 ± 0.001		
		QUIN (μM)	0.053 ± 0.0054*	0.038 ± 0.006		
		PIC (μM)	0.36 ± 0.034	0.51 ± 0.11		
		K/T(×10^3^)	43.7 ± 2	11.1 ± 0.8		

* *P* < 0.05;

** *P* < 0.01;

*** *P* < 0.005;

+ *P* < 0.001;

++ *P* < 0.0001.

**Table 3.** Studies investigating kynurenine metabolites in brain.

**Ref.**	**Pathology**	**Compound**	**Patients**	**Controls**	**Comments**
Beal et al. 1990^40^	HD	KYNA (nM)	1.29 ± 0.14* (HD)	5.10 ± 1.04	PD: Parkinson’s disease; IS: ischemic stroke. 2-fold increase in KYN/KYNA in HD (P < 0.01). KYNA was found to be considerably lower in HD compared to controls and patients with other neurological disorders.
			3.93 ± 0.71 (AD)		
			4.59 ± 1.75 (PD)		
			5.04 ± 1.66 (IS)		
Beal et al. 1992^190^	HD	TRP (ng/g)	4658 ± 442** (i.t.)	8053 ± 1120	p.g.:precentral gyrus; f.c: frontal cortex; i.t.: inferior temporal; m.t: middle temporal; s.t: superior temporal. Kynurenine metabolites, tryptophan, indoleamines and tyrosine and metabolites were anaylzed in 8 different regions of the brain. The data presented here are only for kynurenine metabolites that were significantly different in patients compared to controls.
		KYN (ng/g)	2334 ± 33*** (i.t.)	5884 ± 129	
		KYNA (ng/g)	223 ± 33** (m.t.)	422 ± 83	
		3-HK(ng/g)	18.4 ± 5.3** (p.g.)	81.3 ± 18.1	
			17.9 ± 2.4* (f.c)	31.2 ± 5.6	
			16.0 ± 2.7*** (i.t.)	70.04 ± 17.2	
			17.0 ± 3.7** (m.t.)	39.0 ± 6.4	
			29.4 ± 9.7** (s.t.)	130.3 ± 60.4	
			26.8 ± 8.3** (i.t.)	67.7 ± 19.6	
Pearson and Reynolds. 1992^191^	HDand AD	3-HK(ng/g)	110 ± 47**(HDt.c.)	65 ± 56	t.c: temporal cortex; f.c: frontal cortex; p: putamen In HD, a general increase in 3-HK was observed, rather than a region-specific one. In t.c. of AD cases, where neuronal loss was greater than in HD, suggested that 3-HK increases in HD is not due entirely to neuronal atrophy.
			82 ± 41 (ADt.c.)	65 ± 33	
			93 ± 60**(HDf.c.)	33 ± 26	
			65 ± 47*** (HD p)	19 ± 14	
Sardar et al. 1995^192^	HIV	3-HK(ng/g)	71.3 ± 12.7 (grp)**	19.95 ± 3.18	N-D: HIV without dementia; D: HIV with dementia. Tissues were taken from the frontal cortex. Higher levels of 3-HK in D was not significantly different from N-D. 3-HA formation was an indicator for 3-hydroxykinurease (3-HKase) activity, which was highest in N-D. Thus, increase in 3-HK reflected an overall increase in KP, instead of a decrease in 3-HKase activity.
		3-HA formation (ng/h/g)	64.9 ± 11.4 (N-D)**	15.8 = t2.14	
			85.5 ± 32.8 (D)**		
			66.4 ± 11.5 (grp)**		
			61.6 ± 16.5 (N-D)**		
			75.5 ± 12.5 (D)**		
Heyes et al. 1998^170^	HIV	QUIN (pmol/g)	20942 ± 2959** (bg)	72 ± 26	bg: basal ganglia; wm: cortical white matter; gm: cortical grey matter.
			25397 ± 11435** (wm)	75 ± 12	
			26292 ± 8615** (gm)	81 ± 20	
Bara et al. 2000^193^	HIV	KYN (pmol/mg)	22.66 ± 5.38 (f.c.)	12.08 ± 1.24	f.c: frontal cortex; cb: cerebellum KAT I activity rose significantly in both frontal cortex and cerebellum (341% and 262% of control, respectively), whereas KAT II activity increased only in the frontal cortex (141% of control).
		KYNA (pmol/mg)	24.67 ± 2.62 (cb)	16.33 ± 2.00	
			7.31 ± 1.33(f.c.)	3.49 ± 0.55	
			4.54 ± 0.87 (cb)	2.77 ± 0.63	
Schwarcz et al. 2001^111^	Schizophrenia	KYN (ng/g)	35.2 ± 28.0* (b.a.9)	22.4 ± 14.3	b.a.: Brodmann area KYN, KYNA and 3-HK were tested in b.a 9,10 and 19. Only data that were significantly different from controls are presented here. Positive correlation found between KYN and KYNA but not KYN and 3-HK.
		KYNA (ng/g)	40.3 ± 23.4* (b.a.l9)	30.9 ± 10.8	
			1.9 ± 1.3* (b.a.9)	2.9 ± 2.2	

* *P* < 0.05;

** *P* < 0.01;

*** *P* < 0.005;

+ *P* < 0.001;

++ *P* < 0.0001.
